# SARS-CoV-2 pneumonia and bacterial pneumonia patients differ in a second hit immune response model

**DOI:** 10.1038/s41598-022-17368-9

**Published:** 2022-09-15

**Authors:** Dominique Moser, Matthias Feuerecker, Katharina Biere, Bing Han, Marion Hoerl, Gustav Schelling, Ines Kaufmann, Alexander Choukér, Tobias Woehrle

**Affiliations:** 1 Laboratory of Translational Research ‘Stress and Immunity’, Department of Anesthesiology, Ludwig Maximilians University Hospital, Ludwig-Maximilians-University, Marchioninistr. 15, 81377 Munich, Germany; 2 Department of Anesthesiology, Intensive Care Medicine and Pain Therapy, Municipal Hospitals of Munich, Hospital Neuperlach, Munich, Germany

**Keywords:** Immunology, Diseases

## Abstract

Secondary infections have been shown to complicate the clinical course and worsen the outcome of critically ill patients. Severe Coronavirus Disease 2019 (COVID-19) may be accompanied by a pronounced cytokine release, and immune competence of these patients towards most pathogenic antigens remains uncompromised early in the disease. Patients with bacterial sepsis also exhibit excessive cytokine release with systemic hyper-inflammation, however, typically followed by an anti-inflammatory phase, causing immune paralysis. In a second hit immune response model, leukocyte activation capacity of severely ill patients with pneumonia caused by SARS-CoV-2 or by bacteria were compared upon ICU admission and at days 4 and 7 of the ICU stay. Blood cell count and release of the pro-inflammatory cytokines IL-2, IFNγ and TNF were assessed after whole-blood incubation with the potent immune stimulus pokeweed mitogen (PWM). For comparison, patients with bacterial sepsis not originating from pneumonia, and healthy volunteers were included. Lymphopenia and granulocytosis were less pronounced in COVID-19 patients compared to bacterial sepsis patients. After PWM stimulation, COVID-19 patients showed a reduced release of IFNγ, while IL-2 levels were found similar and TNF levels were increased compared to healthy controls. Interestingly, concentrations of all three cytokines were significantly higher in samples from COVID-19 patients compared to samples from patients with bacterial infection. This fundamental difference in immune competence during a second hit between COVID-19 and sepsis patients may have implications for the selection of immune suppressive or enhancing therapies in personalized medicine.

## Introduction

Severe primary infections can result in high morbidity and mortality. Secondary infections have been shown to further complicate the clinical course and worsen the prognosis, especially in critically ill patients^1–3^. SARS-CoV-2-induced Coronavirus disease 2019 (COVID-19) is frequently accompanied by acute respiratory distress syndrome (ARDS) and may lead to excessive release of pro-inflammatory cytokines, also referred to as cytokine storm^4–7^ which is associated with a worse outcome^4,6,8^. Apart from the life-threatening cytokine storm, we demonstrated in previous investigations that severely ill COVID-19 ARDS patients maintained an immune response similar to healthy control subjects towards *Gram*-positive, *Gram*-negative and *Aspergillus* antigens, but elicited a selectively impaired immune response towards *Candida albicans* in a second hit model^9^.

Similar to the cytokine storm during COVID-19, high concentrations of pro-inflammatory cytokines are released at the onset of bacterial sepsis. This so-called systemic inflammatory response syndrome (SIRS) is a highly inflammatory state with strong abundance of inflammation markers^1,10,11^, which is followed by a compensatory anti-inflammatory response syndrome (CARS), also named immune paralysis^10,12^. Here, the patients´ immune system is ineffective in clearing septic foci and is unable to mount an adequate response against invading pathogens. This leads to an increased susceptibility for secondary infections^13,14^ as demonstrated ex vivo by a blunted innate and adaptive immune response towards a second hit with mitogenic and recall antigen stimulation^10^.

A recent study compared pro-inflammatory cytokine concentrations in severely ill patients suffering from ARDS caused by SARS-CoV-2, from ARDS caused by other pathogens, or from sepsis, upon ICU admission, and cytokine levels did not distinguish between these patient cohorts^15^. However, the differences in ex vivo second hit immune responses observed in COVID-19 patients with ARDS^9^ versus septic patients^10^, both with immune system driven severe clinical complications, raise the question of different activation capacities of leukocytes in these diseases. Thus, we compared the release of the three key adaptive and innate cytokines IL-2, IFNγ and TNF in response to unspecific stimulation with Pokeweed mitogen (PWM) in severely ill COVID-19 patients with pneumonia, in patients with sepsis originating from bacterial pneumonia and in patients with bacterial sepsis originating from other foci than pneumonia. Understanding the differences in general immune competence may have implications for the selection of immune-suppressive or -enhancing therapies in personalized medicine.

## Results

We aimed at investigating differences in the activation capacity of leukocytes from patients with SARS-CoV-2 pneumonia (SARS-P, n = 12) versus bacterial pneumonia (BACT-P, n = 16) in a second hit model. For comparison, we included patients with bacterial sepsis (BACT-S, n = 15) with origin other than pneumonia, and healthy controls (CTRL, n = 11). Detected pathogens are listed in Supplementary Table 1. Stimulation to mimic a second hit was achieved with PWM, and the consecutive release of the pro-inflammatory T cell cytokines IL-2 and IFNγ, and of TNF as a representative cytokine of innate immunity was assessed.

### Study population

Demographic and clinical characteristics of patients and healthy controls are provided in Table 1. Patient cohorts showed no significant difference in age (SARS-P: 64, BACT-P: 62, BACT-S: 71). The CTRL cohort was significantly younger than BACT-S (CTRL: 54, *p* < 0.024). Similarly, gender distribution was similar in SARS-P, BACT-P and CTRL (male sex: 72.7, 81.3 and 72.7%) while BACT-S contained only 33.3% male patients. Mean body mass index (BMI) indicated overweight patients in all cohorts with no difference between groups. Impairment of lung function was found similar in both pneumonia cohorts SARS-P and BACT-P, with a mean PaO_2_/FiO_2_ ratio ranging between 100 and 200, indicating moderate ARDS in both groups. In the BACT-S group, where infection did not originate from the lung, PaO_2_/FiO_2_ ratio was also reduced, with higher median values compared to SARS-CoV-2 and bacterial pneumonia. Determination of SOFA and APACHE II scores revealed a higher disease severity in BACT-P and BACT-S compared to SARS-P, while the 4C mortality score for SARS-P confirmed high severity of disease and a high median mortality risk of 31.4% (31.4–61.6%)^16^. Duration of ventilation, length of ICU stay and overall hospitalization did not show significant differences between SARS-P and BACT-P, while BACT-S required a significantly shorter duration of ventilation and shorter ICU stay compared to both pneumonia cohorts. No SARS-CoV-2 patient died during the evaluated interval, and mortality rates of patients with bacterial pneumonia and bacterial sepsis were similar (Table 1 and Supplementary Table 2).

**Table 1:** Demographic characterization of patients with SARS-CoV-2 pneumonia (SARS-P, n = 12), bacterial pneumonia (BACT-P, n = 16), bacterial sepsis with origin other than pneumonia (BACT-S, n = 15) and of healthy control subjects (CTRL, n = 11).

Characteristic	SARS-P	BACT-P	BACT-S	CTRL	*p*-value	*p*-value
					SARS-P versus BACT-P	SARS-P versus BACT-S
Age (years)	64 (57–72)	62 (50–74)	71 (59–76)	54 (49–62)	.456^a^	**.032**^**a**^
Male sex (%)	8 (72.7)	13 (81.3)	5 (33.3)	8 (72.7)		
BMI (kg/m^2^)	27.7 (25.1–31.1)	27.0 (24.1–35.1)	29.0 (24.7–32.9)	25.3 (23.8–28.2)	.902^b^	.671^a^
**PaO**_**2**_**/FiO**_**2**_** ratio**						
Day 0	107 (91–174)	115 (62–179)	152 (114–218)	n.a	.622^b^	.223^a^
Day 4	182 (138–190)	150 (74–173)	205 (113–264)	n.a	.508^a^	.308^a^
Day 7	199 (181–234)	166 (120–213)	140 (62–214)	n.a	.287^a^	.063^a^
**Severity scores (ICU admission)**						
SOFA	10 (7.5–11.5)	15 (14–16.8)	14 (11–16.5)	n.a	** < .001**^**a**^	**.009**^**b**^
APACHE II	24.5 (18.6–26.8)	29.5 (23–37)	30.5 (23.5–33)	n.a	**.023**^**b**^	**.021**^**a**^
4C	14 (12–16)	n.a	n.a	n.a		
**Days**						
Ventilated	15 (11.5–22.5)	12 (9.3–18.0)	2 (0–13)	n.a	.374^b^	**.008**^**b**^
On ICU	23 (14.5–28)	20 (15–37.5)	8 (3–19)	n.a	.863^b^	**.032**^**b**^
In hospital	34 (25.5–47)	23.5 (15–54.8)	30 (26–50)	n.a	.855^a^	.954^b^
**Mortality, n (%)**						
On ICU	0 (0)	3 (18.8)	3 (20.0)	n.a		
At day 28	0 (0)	2 (12.5)	2 (13.3)	n.a		

Significant values are in [bold].

*SOFA* Sequential Organ Failure Assessment, *APACHE II* Acute Physiology And Chronic Health Evaluation II, *4C* Coronavirus Clinical Characterisation Consortium-Mortality Score.

Values are median (IQR).

^a^two-tailed unpaired Student’s *t*-test, ^b^Mann–Whitney-*U* test.

Additionally, patients were characterized by routinely measured blood cell counts and inflammation markers (Table 2). Total leukocyte counts showed no statistical difference between cohorts, but median counts were higher in cohorts with bacterial infection, compared to SARS-P. A similar pattern was observed for total monocyte counts. Compared to BACT-P, SARS-P showed a significantly elevated lymphocyte count, while the percentage of neutrophils was decreased. Thrombocyte counts in SARS-P were found elevated compared to both cohorts with bacterial infection. Erythrocyte counts and hemoglobin did not differ between groups (Table 2 and Supplementary Table 2). The inflammation markers PCT and IL-6 were significantly higher in both bacterial cohorts compared to SARS-P, while levels of CRP did not show significant differences (Table 2 and Supplementary Table 2).

**Table 2:** Blood cell count and proinflammatory markers of patients with SARS-CoV-2 pneumonia (SARS-P, n = 12), bacterial pneumonia (BACT-P, n = 16), bacterial sepsis with origin other than pneumonia (BACT-S, n = 15) and of healthy control subjects (CTRL, n = 11).

Characteristic	SARS-P	BACT-P	BACT-S	CTRL (reference range)	*p*-value	*p*-value
					SARS-P versus BACT-P	SARS-P versus BACT-S
Leukocytes (cells/µl)	10,600 (7530–12,400)	13,400 (6025–21,200)	17,400 (6450–28,450)	4000–11,000	.456^a^	**.032**^**a**^
Lymphocytes (cells/µl)	1090 (764–1316)	695 (360–1157)	985 (239–2174)	900–3500	**.033**^**a**^	.751^b^
(%)	11 (9–16)	5.5 (2.3–8.5)	7 (3.8–12.3)	22–45	**.003**^**b**^	.066^b^
Monocytes (cells/µl)	322 (202–628)	746 (49–1238)	1038 (176–1198)	280–900	.228^a^	.130^b^
(%)	4 (3–7)	3.5 (2–7.8)	5 (3–7.3)	4–12	.765^b^	.929^a^
Neutrophils (cells/µl)	7530 (6230–9980)	9743 (5280–17,340)	14,280 (5758–20,634)	1700–7000	.246^b^	.113^b^
(%)	74 (64–82)	85 (77–91)	81 (63–88)	40–70	**.025**^**b**^	.307^b^
Thrombocytes (× 10^9^/L)	336 (241–387)	169 (89–232)	181 (67–257)	146–328	** < .001**^**a**^	**.008**^**a**^
Erythrocytes (× 10^12^/L)	4.01 (3.17–4.22)	2.94 (2.88–3.50)	3.14 (2.94–3.48)	4.5–6.0	.076^a^	.147^a^
Hemoglobin (g/dl)	11.5 (9.8–12.3)	10.0 (9.5–11.0)	9.2 (8.4–10.7)	11.5–15.4	.288^b^	.130^a^
PCT (ng/ml)	0.9 (0.3–1.2)	12 (7.3–45.9)	36.5(8.8–70.2)	< 0.1 ng/ml	** < .001**^**b**^	** < .001**^**b**^
CRP (mg/dl)	22.3 (10.2–30.7)	27.8 (17.8–32.6)	21.0 (12.7–27.1)	< 0.5 mg/dl	.104^a^	.888^a^
IL-6 (pg/ml)	333 (58.6–603)	1446 (221–11,972)	3654 (2142–20,698)	< 5.9 pg/ml	**.036**^**a**^	** < .001**^**a**^

Significant values are in [bold].

Values are median (IQR).

^a^two-tailed unpaired Student’s *t*-test, ^b^Mann–Whitney-*U* test.

In summary, patient cohorts were similar with respect to their demographics, and leukocyte subpopulations showed characteristic differences for viral versus bacterial infections.

### Ex vivo immune response after PWM stimulation

In order to assess potential differences in the overall leukocyte activation capacity between patients with SARS-CoV-2 pneumonia or bacterial pneumonia, levels of IL-2, IFNy and TNF were determined after ex vivo stimulation with PWM, or in vehicle treated controls in whole blood samples drawn on the day of ICU admission (day 0), and potential changes over time were assessed in samples obtained on day 4 and day 7. Patients with bacterial sepsis and healthy volunteers were included as control cohorts.

### IL-2 secretion in whole blood incubation assay

**Figure 1:**
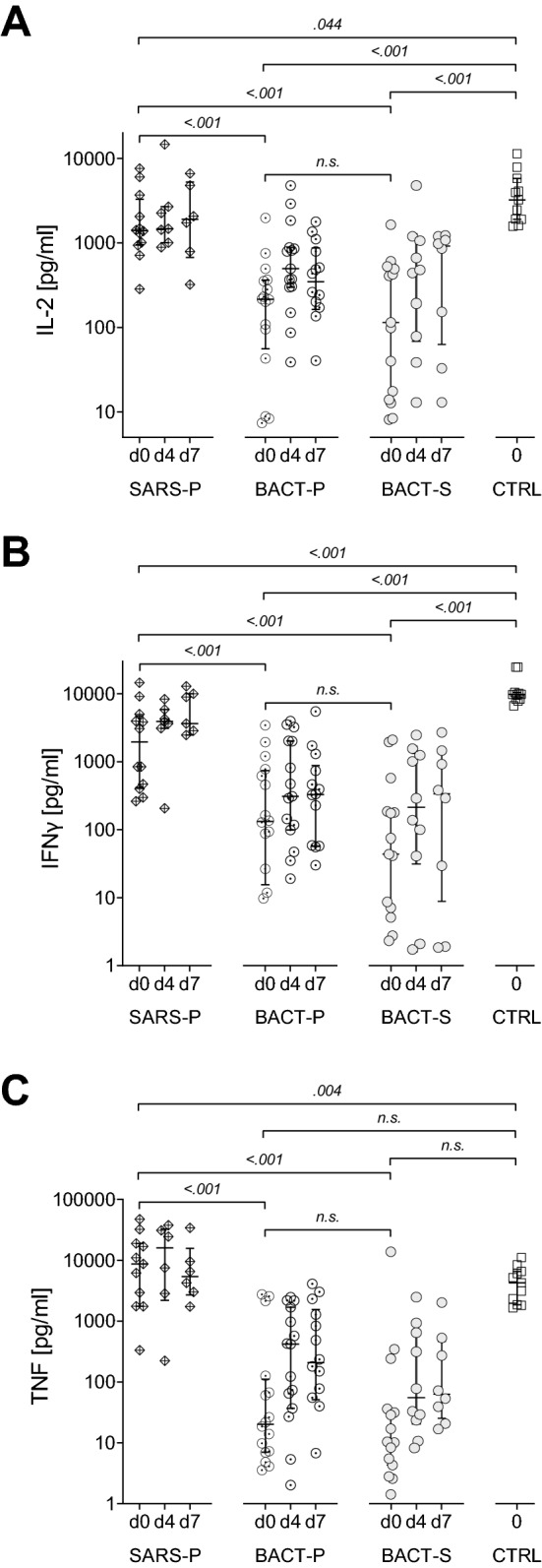
Cytokine concentrations after stimulation with pokeweed mitogen (PWM). Concentrations of IL-2 (**A**), IFN-γ (**B**) and TNF (**C**) were measured in whole blood samples obtained from patients with severe COVID-19 (SARS-P, d0: n = 12, d4: n = 7, d7: n = 7), from patients with sepsis resulting from bacterial pneumonia (BACT-P, d0: n = 16, d4: n = 15, d7: n = 14), from patients with sepsis resulting from bacterial origin other than pneumonia (BACT-S, d0: n = 15, d4: n = 10, d7: n = 8) and from healthy volunteers (CTRL, n = 11), after PWM stimulation (5 µg/ml) for 48 h at day 0, day 4 and day 7. *n.s.*; non-significant, two-way ANOVA and Holm-Šídák test.

After PWM stimulation on day 0, levels of IL-2 were found significantly reduced in all three patient cohorts compared to CTRL. However, IL-2 release in SARS-P was significantly higher than in cohorts with bacterial infection, where concentrations did not differ between BACT-P and BACT-S. On day 4 and day 7, neither SARS-P nor BACT-P or BACT-S showed differences in IL-2 release capacity compared to day 0 (Fig. 1A). In vehicle control samples, IL-2 was low in all cohorts, with no differences between groups or time points analyzed (Supplementary Table 4).

### IFNy secretion in whole blood incubation assay

Compared to CTRL, IFNy levels were significantly decreased in all patient cohorts after PWM stimulation (Fig. 1B). Similar to patterns observed for IL-2, SARS-P displayed significantly higher IFNy levels than both bacterial cohorts, and while median values further increased, no significant difference in IFNy levels was observed on day 4 and day 7. In samples from BACT-P and BACT-S, IFNy concentrations remained unaltered on days 4 and 7. Vehicle control samples showed no difference between patient cohorts, with higher IFNy levels in unstimulated CTRL samples (Supplementary Table 4).

### TNF secretion in whole blood incubation assay

Compared to SARS-P, and in line with results obtained for IL-2 and IFNy, both cohorts with bacterial infection displayed significantly lower TNF levels on all three days. For CTRL, median TNF concentrations were higher than in BACT-P and BACT-S, however, without reaching statistical significance. Interestingly, we observed higher TNF levels after PWM stimulation in the SARS-P group on day 0 compared to CTRL. Both bacterial cohorts showed higher median values on days 4 and 7, compared to day 0, with no statistical difference between these days (Fig. 1C). Remarkably, vehicle control samples also showed significantly increased basal TNF levels in SARS-P, compared to both bacterial cohorts and CTRL (Supplementary Tables 3 and 4).

In addition to the unspecific stimulation with PMW, we also used *Aspergillus fumigatus* lysate in samples obtained on day 0 to assess potential differences in leukocyte activation by this clinically relevant pathogen. Median IL-2 levels in response to *Aspergillus fumigatus* were significantly lower in BACT-P and BACT-S compared to CTRL, while there was no significant reduction in SARS-P. Stimulation-induced IFNγ release remained low in all cohorts with no differences between groups. Concentrations of TNF were found significantly enhanced in SARS-P compared to BACT-P, BACT-S and CTRL, similar to incubation with PWM (Supplementary Fig. 1).

## Discussion

In the present study, we compared general leukocyte activation capacities in patients with ARDS resulting either from SARS-CoV-2 or from bacterial pneumonia. For comparison, patients with bacterial sepsis without pneumonia and healthy volunteers were included. We performed ex vivo whole blood stimulation assays with subsequent analyses of the resulting cytokine profile which reflects the patient’s ability to mount an immune response against pathogens. Specifically, immune responses were analyzed by secretion of the key innate and adaptive cytokines IL-2, IFNγ and TNF after stimulation with PWM.

Results indicate that upon admission to the ICU and over the course of 7 days, leukocytes from patients with severe COVID-19 maintained their ability for a pronounced release of IL-2, IFNy and TNF in response to PWM, while leukocytes from patients suffering from severe bacterial infections showed an impaired release of these cytokines. Interestingly, while SARS-CoV-2 patients elicited similar or lower levels for the T cell cytokines IL-2 and IFNy compared to healthy controls, the release of TNF as a representative of innate immune cell cytokines was found to be increased in most SARS-CoV-2 patients, indicating macrophage activation syndrome^17^. In contrast, septic patients displayed irrespective of presence or absence of pneumonia only low secretion levels of these cytokines in comparison to healthy controls and COVID-19 patients.

One immunological hallmark of sepsis is the systemic inflammatory response syndrome (SIRS) which is associated with cytokine storm and potentially results in tissue damage and ultimately organ failure^11,18^. This initial hyper-inflammatory phase is followed by a compensatory anti-inflammatory response syndrome (CARS) within 24 h to limit excessive organ damage^10,19^. In this state, the immune system is compromised in counteracting the initial infection, which is accompanied by an increased susceptibility for secondary infections, causing high number of deaths in the later phase of sepsis^10,13,14,19^. Assumed reasons for CARS are direct apoptosis of dendritic cells, B cells and CD4^+^ and CD8^+^ T cells, a higher proportion of T_regs_, that have shown to be less vulnerable to sepsis-induced apoptosis and to enhance anti-inflammatory regulation, and a deviation from T_h_1-dominated initial pro-inflammatory response to a T_h_2-dominated anti-inflammatory immune response^13,19,20^.

COVID-19 is likewise characterized by a hyper-inflammatory cytokine pattern and secretion intensity is associated with an unfavorable prognosis^4,6,8^. Here, the observed cytokine storm is mainly attributed to macrophage activation syndrome^9,17,21^. However, this hyper-inflammatory phase is not followed by a compensatory immune suppression but rather by an ongoing inflammation due to cellular release of damage associated molecular patterns (DAMPs)^22,23^. DAMPs, which are released in response to inflammation-induced tissue damage are primarily taken up by macrophages, which leads to further activation and an ongoing hyper-inflammatory state, driven mainly by the innate immune system^23,24^. Like patients suffering from severe bacterial infection, COVID-19 patients in this study showed a marked granulocytosis, however to a lesser extent than patients with bacterial pneumonia, which is known to be associated with a severe clinical course for both disease patterns^25,26^. Both SOFA and APACHE II scores indicated a lower disease severity in COVID-19 patients compared to bacterial sepsis. This can be attributed to the pathology of the 2020 SARS-CoV-2 variants, which affected the lung as the main and primary organ, leading to the necessity of mechanical ventilation at an early stage. Bacterial infections and their cellular components may affect additional organs quicker than SARS-CoV-2 and thus result in higher SOFA and APACHE II scores. Nonethess, for COVID-19 patients examined in this study, the 4C score demonstrated high severity of disease and high risk of in-hospital mortality.

Interestingly, in this COVID-19 patient cohort, a disease-associated immune paralysis and inability to react towards antigen stimulation as observed for the bacterial sepsis cohort and as reported for other COVID-19 patients^27,28^ did not occur. Exclusively PWM-induced IFNy secretion was reduced compared to healthy controls. Low plasmatic levels of type I and II interferons were detected in a considerable proportion of critically ill COVID-19 patients, which prevents an antiviral immune response and is connected to a poor outcome^29^, where virally driven hyperinflammation is the main cause for mortality^30,31^. Defects in production of IFNy after antigenic stimulation were also documented in COVID-19 patients^9,32^, which most likely can be attributed to depletion of IFNy producing cells^32^. Nevertheless, in the present investigation, the ex vivo IFNy response in COVID-19 patients still exceeded the response of sepsis patients.

Together with the observed IL-2 and TNF levels, these findings indicate a maintained multifunctional immune capacity during COVID-19 in comparison to patients with septic bacterial pneumonia and bacterial sepsis in the early phase of the ICU stay, and suggest different impacts of these two disease patterns on immunity.

Recent reports addressing the incidence of secondary infections in severe COVID-19 patients are controversial. While a considerable amount of retrospective reviews and meta-analyses show that less than 8% of hospitalized COVID-19 patients suffer from secondary microbial infections^33–37^, other reports indicate higher numbers of secondary infections in these patients, such as ventilator-associated pneumonias^38–41^. Recent literature suggests that in these patients, increased susceptibility towards microbial superinfection is associated with critical care^42,43^ and with lesions of pulmonary tissue^44,45^ which favor microbial colonization, rather than exclusively with an impaired immune response^46^. This is further corroborated by the pronounced immune response to *Aspergillus fumigatus* presented in this study, where no secondary infections were observed up to day 7, while other reports describe pulmonary aspergillosis to be associated with COVID-19^47,48^. In contrast, occurence of bacterial or fungal secondary infections in sepsis patients correlates with disease severity^2^ and is reported strikingly above 10%, with the highest susceptibility towards *Candida spp.* and increasing infection rates in the later phase of the disease^2,49^.

Changes on the molecular and protein level that might contribute to an impaired function of the immune system, such as cell surface expression of HLA-DR on monocytes (mHLA-DR), were not evaluated in the present study. Downregulation of mHLA-DR is an established marker for monocyte suppression and disease severity in sepsis^50,51^ and has been reported to correlate with disease severity in COVID-19^52,53^. However, mHLA-DR levels in this present COVID-19 patient cohort were comparable to healthy controls in previous investigations^9^, further underlining the functionality of their monocytes. Besides a variety of other potential but yet unexplored differences on the cellular level of leukocytes in COVID-19 and sepsis, the detected differences in the extent of granulocytosis and lymphopenia may contribute to the observed immune response.

## Considerations and limitations

We acknowledge the limitations of this investigation, related to the analysis of the effects of only two antigens (PWM and *A. fumigatus*) and the low number of analyzed cytokines. We also acknowledge that comparisons of other relevant immune functions such as NETosis or ROS production as well as detailed analysis on cell populations which may significantly contribute to the disease course and susceptibility towards nosocomial infections were beyond the scope of this study. The investigations of both COVID-19 and sepsis patient cohorts were conducted as single center studies by the same institution, and measurements were conducted with similar assays. This allowed for comparison of leukocyte activation capacities in viral and bacterial disease, which would not have been possible during the COVID-19 pandemic due to a lack of bacterial pneumonia patients at our institution. In addition, this analysis represents a new starting point for a detailed examination of differential immune responses among severe systemic inflammatory syndromes, which may contribute to an appropriate consideration of personalized immunomodulatory therapies.

## Materials and methods

### Study populations

Between April and May 2020, patients with pneumonia resulting from confirmed SARS-CoV-2 infection were included in this study upon admission to the ICU, when they required intubation and ventilation. All COVID-19 patients received treatment according to the standard COVID-19 treatment protocol as of April 2020^9^. Arterial blood was drawn on the day of ICU admission (day 0; n = 12) and on day 4 (n = 7) and day 7 (n = 7) of the ICU stay, and the patients’ detailed clinical course was recorded. Data from patients with sepsis resulting from bacterial pneumonia (d0: n = 16, d4 and d7: n = 15) and from patients with bacterial sepsis without pneumonia (d0: n = 15, d4: 10–12 and d7: n = 8–12) were recorded as part of the prospective, randomized, multicenter clinical trial “Placebo Controlled Trial of Sodium Selenite and Procalcitonin Guided Antimicrobial Therapy in Severe Sepsis” (SISPCT, NCT00832039) from June 2011 to February 2013^10,54^. Reduction in patient numbers from d0 to d4 and d7 was due to discharge of the ICU, withdrawal of consent, or death. Patients with a malignant disease, autoimmune disorder or previous immunosuppressive therapy were excluded. Healthy volunteers (n = 11) who were recruited between September and December 2012 served as control cohort^10^.

### Blood processing

Complete Blood Count (CBC): Erythrocyte counts, leukocyte subpopulations and platelet counts as well as hemoglobin concentrations were assessed upon admission at the intensive care unit (ICU) (Institute of Laboratory Medicine, University of Munich, Germany).

#### Plasma Inflammation Markers

C-reactive protein (CRP), Interleukin-6 (IL-6) and procalcitonin (PCT) were routinely assessed and measured according to standard procedures (Institute of Laboratory Medicine, University of Munich, Germany).

#### Ex Vivo Whole Blood Stimulation Assay

Lithium-heparin whole blood was diluted with an equal volume of RPMI 1640 (Sigma-Aldrich, Steinheim, Germany) for functional analysis in COVID-19 patients and DMEM (Dulbecco´s Modifed Eagle’s Medium Nutrient Mixture F-12 HAM, Sigma-Aldrich, Steinheim, Germany) for sepsis patients and healthy controls and stimulated with the potent immune activator and B- and T cell mitogen Pokeweed mitogen (PWM; 5 µg/ml; Sigma-Aldrich, Steinheim, Germany)^55^, *Aspergillus fumigatus* lysate (10 μg/ml; Raybiotech, Georgia, USA) or incubated with vehicle only as negative control. Samples were incubated for 48 h at 37 °C. Following incubation, supernatants were collected and stored at − 80 °C until cytokine measurement.

### Cytokine measurement

Concentration of the cytokines IL-2, IFNγ and TNF from thawed ex vivo incubation assay supernatants were quantified using bead-based Multiplex assays with MAGPIX Multiplexing System (Luminex, Austin, TX, USA) for COVID-19 patients in 2020 and Luminex xMAP® technology (Bioplex®) for bacterial pneumonia and sepsis patients in 2013 with commercially available reagents from BioRad-Laboratories Inc. (California, USA). A standard curve was obtained from the same standard dilution intervals.

### Statistical analysis

Data analysis was performed with commercially available software (SigmaPlot 12.5, Systat, Erkrath, Germany; GraphPad Prism 8.1.1, San Diego, CA, USA). Unless otherwise stated, results are expressed as median (IQR). For comparison between two groups, two-tailed unpaired Student’s *t*-test or Mann–Whitney-*U* test were used. For multiple pairwise comparisons, two-way ANOVA and the Holm-Šídák test were applied. Differences were considered significant at *p* < 0.05.

### Study Approval

Informed consent was obtained from the patients, next of kins or legal representatives and from healthy volunteers, respectively. The studies were performed after obtaining the LMU Medical Faculty ethics committee approval (#20-271) and Ethical study approval for additional experiments in the scope of the *immune function study* part as a local amendment to the approved SISPCT study (Eudra-CT-Nr. 2007-004333-42)^54^ from the ethical board of the University of Jena, Germany. In case of withdrawal, the patient was immediately excluded from the study and no further follow-up was performed. The studies were conducted in agreement with the ethical norms and standards of the Declaration of Helsinki.

## Supplementary Information

Supplementary Information.

## Data Availability

Supplementary information accompanies this manuscript and is attached as single file.
