# Supplementary material for: SARS-CoV-2 pneumonia and bacterial pneumonia patients differ in a second hit immune response model
Source: Sci Rep. 2022 Sep 15;12:15485. doi: 10.1038/s41598-022-17368-9 (PMC9476429; doi:10.1038/s41598-022-17368-9)
Supplement: Supplementary file 1 — Supplementary Information. [file 41598_2022_17368_MOESM1_ESM.pdf]

SARS-CoV-2 pneumonia and bacterial pneumonia patients differ in a second hit immune response model

Dominique Moser, Matthias Feuerecker, Katharina Biere, Bing Han, Marion Hoerl, Gustav Schelling, Ines Kaufmann, Alexander Choukér and Tobias Woehrle

Supplementary Information

**Pathogen**	**SARS-P**	**BACT-P**	**BACT-S**
**Virus**			
SARS-CoV-2	11	0	0
***Gram*-negative bacteria**			
Legionella pneumophila		4	0
Clamydia pneumoniae		1	0
Pseudomonas aeruginosa		2	1
Serratia		1	0
Escherichia coli		1	5
Klebsiella		1	2
***Gram*-positive bacteria**			
Streptococcus pneumoniae		4	0
Streptococcus spp.		0	1
Staphylococcus spp.		2	0
Staphlococcus koag.-neg		0	4
Enterococcus faecium		0	1
Clostridium difficile		0	1

Supplementary Table 1. Detected pathogens in sepsis patients with and without pneumonia.

	SARS-P vs. BACT-P	SARS-P vs. BACT-S	BACT-P vs. BACT-S	SARS-P vs. CTRL	BACT-P vs. CTRL	BACT-S vs. CTRL
Characterization of patients and healthy controls						
**Age (years)**	.456[a]	.32[a]	.107[a]	.139[a]	.566[a]	**.024**[a]
**BMI (kg/m^2)**	.902[b]	.671[a]	.768[a]	.237[b]	.43[b]	.196[b]
**PaO_2/FiO_2 ratio day 0**	.622[b]	.223[a]	**.039**[b]	n.a.	n.a.	n.a.
**Severity scores**						
SOFA	**<.001**[a]	**.009**[b]	.439[b]	n.a.	n.a.	n.a.
APACHE II	**.023**[b]	**.021**[a]	.79[a]	n.a.	n.a.	n.a.
**Days**						
ventilated	.374[b]	**.008**[b]	**.011**[b]	n.a.	n.a.	n.a.
on ICU	.863[b]	**.032**[b]	**.013**[b]	n.a.	n.a.	n.a.
in hospital	.855[a]	.954[b]	.725[b]	n.a.	n.a.	n.a.
Blood counts						
**Leukocytes (cells/µl)**	.208[b]	.183[b]	.644[a]	n.a.	n.a.	n.a.
**Lymphocytes (cells/µl)**	**.033**[a]	.751[b]	.414[b]	n.a.	n.a.	n.a.
(%)	**.003**[b]	.066[b]	.508[b]	n.a.	n.a.	n.a.
**Monocytes (cells/µl)**	.228[a]	.13[b]	.492[a]	n.a.	n.a.	n.a.
(%)	.765[b]	.929[a]	**.044**[b]	n.a.	n.a.	n.a.
**Neutrophils (cells/µl)**	.246[b]	.113[b]	.699[a]	n.a.	n.a.	n.a.
(%)	**.025**[b]	.307[b]	.279[b]	n.a.	n.a.	n.a.
**Thrombocytes (×10^9/L)**	**<.001**[a]	**.008**[a]	.851[a]	n.a.	n.a.	n.a.
**Erythrocytes (×10^{12}/L)**	.076[a]	.147[a]	.509[b]	n.a.	n.a.	n.a.
**Hemoglobin (g/dl)**	.288[b]	.13[a]	.174[b]	n.a.	n.a.	n.a.
**PCT (ng/ml)**	**<.001**[b]	**<.001**[b]	.336[b]	n.a.	n.a.	n.a.
**CRP (mg/dl)**	.104[a]	.888[a]	.051[a]	n.a.	n.a.	n.a.
**IL-6 (pg/ml)**	**.036**[b]	**.001**[b]	.119[b]	n.a.	n.a.	n.a.

Supplementary Table 2. Results of statistical analyses *Characterization of patients and healthy controls* between all cohorts; [a]two-tailed unpaired Student's *t*-test, [b]Mann–Whitney-*U* test.

		SARS-P vs. BACT-P	SARS-P vs. BACT-S	BACT-P vs. BACT-S	SARS-P vs. CTRL	BACT-P vs. CTRL	BACT-S vs. CTRL
Cytokine concentrations							
Negative control samples							
**IL-2**	day 0	n.a.	n.a.	.967[b]	n.a.	.24[b]	.496[b]
**IFNγ**	day 0	.385[b]	.335[b]	.342[b]	**.03[b]**	.415[b]	**.004[b]**
**TNF**	day 0	**<.001[b]**	**<.001[b]**	.826[b]	**<.001[b]**	.98[b]	.769[b]
PWM stimulated samples (two-way ANOVA)							
**IL-2**	day 0	**<.001**	**<.001**	.975	**.044**	**<.001**	**<.001**
**IFNγ**	day 0	**<.001**	**<.001**	.745	**<.001**	**<.001**	**<.001**
**TNF**	day 0	**<.001**	**<.001**	.959	**.004**	.258	.189

Negative control samples over time

		SARS-P	BACT-P	BACT-S
**IL-2**	day 0 vs. day 4	.947	.068	.15
	day 0 vs. day 7	.947	.983	.973
	day 4 vs. day 7	1	.068	.183
**IFNγ**	day 0 vs. day 4	1	.077	.191
	day 0 vs. day 7	.526	.267	.77
	day 4 vs. day 7	.62	.739	.823
**TNF**	day 0 vs. day 4	.808	.952	.394
	day 0 vs. day 7	.658	.949	1
	day 4 vs. day 7	.886	.877	.68

PWM stimulated samples over time (two-way ANOVA)

		SARS-P	BACT-P	BACT-S
**IL-2**	day 0 vs. day 4	n.s.	n.s.	n.s.
	day 0 vs. day 7	n.s.	n.s.	n.s.
	day 4 vs. day 7	n.s.	n.s.	n.s.
**IFNγ**	day 0 vs. day 4	n.s.	n.s.	n.s.
	day 0 vs. day 7	n.s.	n.s.	n.s.
	day 4 vs. day 7	n.s.	n.s.	n.s.
**TNF**	day 0 vs. day 4	n.s.	n.s.	n.s.
	day 0 vs. day 7	n.s.	n.s.	n.s.
	day 4 vs. day 7	n.s.	n.s.	n.s.

Supplementary Table 3. Results of statistical analyses *Cytokine concentrations* between all cohorts; [a]two-tailed unpaired Student's *t*-test, [b]Mann–Whitney-*U* test, for multiple pairwise comparisons, two-way ANOVA and the Holm-Šídák test were applied.

		SARS-P	BACT-P	BACT-S	CTRL
**IL-2 [pg/ml]** median (IQR)	day 0	< LOD	0.1 (0.0-0.7)	0.1 (0.0-1.3)	0.3 (0.1-1.13)
	day 4	< LOD	< LOD	< LOD	n.a.
	day 7	< LOD	0.1 (0.0-1.5)	0.1 (0.0-1.2)	n.a.
**IFNγ [pg/ml]** median (IQR)	day 0	0.4 (0.40-7.3)	3.0 (0.4-53.1)	2.8 (0.5-6.2)	11.7 (3.4-16.6)
	day 4	0.4 (0.4-1.9)	0.4 (0.2-2.0)	1.5 (0.3-1.6)	n.a.
	day 7	0.4 (0.4 -4.2)	0.8 (0.0-23.0)	0.8 (0.2-16.9)	n.a.
**TNF [pg/ml]** median (IQR)	day 0	180 (95-317)	0.3 (0.1-2.0)	0.3 (0.0-3.2)	0.7 (0.0-8.4)
	day 4	166 (78-663)	0.5 (0.2-1.6)	0.3 (0.0-0.9)	n.a.
	day 7	194 (57-498)	0.6 (0.0-7.7)	0.4 (0.0-3.6)	n.a.

Supplementary Table 4. Concentrations of IL-2, IFN-γ and TNF were measured in whole blood samples obtained from patients with severe COVID-19 (SARS-P, n=12), patients with sepsis resulting from bacterial pneumonia (BACT-P, n=16), from patients with sepsis resulting from bacterial origin other than pneumonia (BACT-S, n=15) and from healthy volunteers (CTRL, n=11), after vehicle control stimulation for 48 hours on day 0, day 4 and day 7.

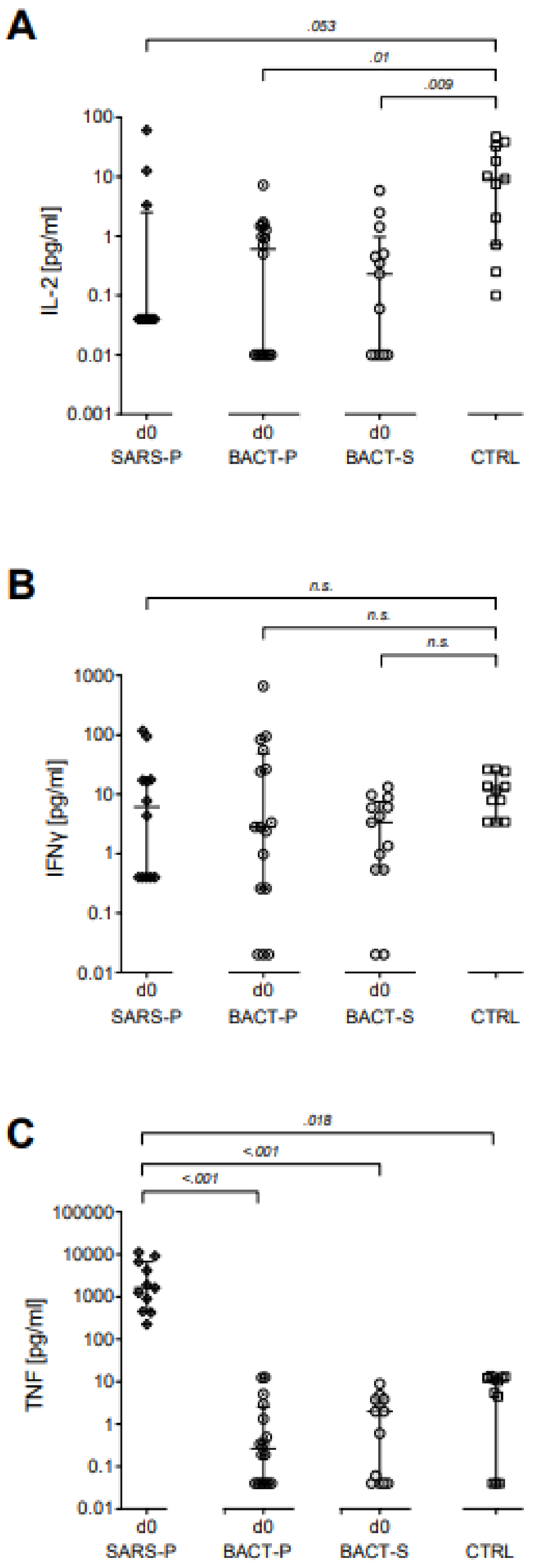

Supplementary Figure 1. Cytokine concentrations after stimulation with *Aspergillus fumigatus* lysate. Concentrations of IL-2 (**A**), IFN-γ (**B**) and TNF (**C**) were measured in whole blood samples obtained from patients with severe COVID-19 (SARS-P, n=12), patients with sepsis resulting from bacterial pneumonia (BACT-P, d0: n=16), from patients with sepsis resulting from bacterial origin other than pneumonia (BACT-S, d0: n=13) and from healthy volunteers (CTRL, n=11), after *Aspergillus fumigatus* stimulation (10 µg/ml) for 48 hours upon ICU admission; *n.s.* non-significant, data were analyzed using one-way ANOVA.
